# Development of a Monoclonal Antibody Against Porcine CD163 SRCR5 Domain Which Partially Blocks Infection of PRRSV

**DOI:** 10.3389/fvets.2020.597843

**Published:** 2020-11-05

**Authors:** Yujiao Zhang, Kuan Zhang, Hao Zheng, Changlong Liu, Yifeng Jiang, Nannan Du, Liwei Li, Guoxin Li, Lingxue Yu, Yanjun Zhou, Wu Tong, Kuan Zhao, Guangzhi Tong, Fei Gao

**Affiliations:** ^1^Department of Swine Infectious Diseases, Shanghai Veterinary Research Institute, Chinese Academy of Agricultural Sciences, Shanghai, China; ^2^Jiangsu Co-innovation Center for the Prevention and Control of Important Animal Infectious Disease and Zoonosis, Yangzhou University, Yangzhou, China; ^3^College of Animal Science, Fujian Agriculture and Forestry University, Fuzhou, China

**Keywords:** PRRSV, epitope, SRCR5, CD163, monoclonal antibody

## Abstract

Porcine reproductive and respiratory syndrome virus (PRRSV), which seriously endangers the world pig industry, invades host cells through receptor-mediated endocytosis involving clathrin. CD163 is an essential receptor for PRRSV during its infection of cells. The scavenger receptor cysteine-rich 5 (SRCR5) domain of the CD163 molecule is necessary for PRRSV infection, and interacts with glycoproteins GP2a and GP4 of PRRSV, allowing the virus to infect the host cells. In this study, a monoclonal antibody (mAb) against the SRCR5-6 region of porcine CD163 was developed, and the target epitope of the mAb was determined as ^497^TWGTVCDSDF^506^, which is directly adjacent to the ligand-binding pocket (LBP) domain (487-495aa) of CD163. Further study indicated that the mAb could partially block PRRSV infection of its target cells, pulmonary alveolar macrophages. The mAb developed in the study may provide a foundation of antiviral therapy for PRRSV.

## Introduction

CD163 is a member of the scavenger receptor cysteine-rich (SRCR) family, and is mainly expressed on monocytes, macrophages (1), and specific subsets of dendritic cells (2). CD163, as a receptor that scavenges hemoglobin by mediating the endocytosis of haptoglobin-hemoglobin complexes during haemolysis (3) and also plays a role as an innate bacterial immune sensor, inducing pro-inflammatory cytokine production (4). Soluble CD163 is considered as a marker of macrophage activation and is strongly associated with Human immunodeficiency virus (HIV) and Hepatitis C virus infections (5). The abundance of CD163 is closely related to HIV infection, and the productivity of HIV infection was higher in CD163 abundant cells, whereas a significantly weaker HIV infection was observed in CD163-knocked-down macrophages (6).

The interaction between CD163 and the virus is particularly important, and has been extensively studied in relation to Porcine reproductive and respiratory syndrome virus (PRRSV) (7) and African swine fever virus (8), both of which have devastating impacts on the swine industry.

PRRSV is a major pathogen that seriously harms swine herds, causing immense economic losses to swine industry worldwide. Porcine reproductive and respiratory syndrome is characterized by late-term reproductive failure in sows and severe pneumonia in neonatal pigs. The PRRSV particle is enveloped and the viral genome is a single-stranded positive-sense RNA molecule. Alveolar macrophages (AMs) are the target cells of PRRSV, CD163 on porcine AM membranes has been revealed as an essential receptor mediating infection of PRRSV (9). CD163, occurs specifically on the surfaces of monocytes and macrophages and is a type I transmembrane protein. It has a molecular weight of about 130 kDa. It contains an extracellular region, transmembrane region, and intracellular region. Its extracellular domain contains nine cysteine-rich domains and two proline-serine-threonine-rich (PST) motifs (10). After the transient or stable expression of CD163 molecules, two PRRSV-impermissible cell lines supported both type I and type II PRRSV infections and produced progeny viral particles, indicating that CD163 plays a key role in promoting viral uncoating and the release of genomic RNA into the cytoplasm. During PRRSV infection, CD163 interacts with the glycosylated membrane proteins GP2a and GP4 of PRRSV to facilitate the replication of the virus (7). In the cell, CD163 co-locates with the N protein of PRRSV in early endosomes, but not in late endosomes (11). Further studies showed that the SRCR5 domain of the CD163 extracellular region plays a key role in PRRSV infection, and pig deleted SRCR5 of CD163 can fully resist the infection of PRRSV (12). Ma et al. (13) found that the R561 of CD163 SRCR5 has impact on PRRSV replication. In this study, the extracellular region of CD163 protein, SRCR5-SRCR6 was expressed and purified in a prokaryote and used to immunize BALB/c mice, to prepare a mouse anti-pig CD163 monoclonal antibody (mAb). The epitope ^497^TWGTVCDSDF^506^ was identified, which is very adjacent to the LBP domain of CD163. The antibody can partly block the replication of PRRSV, confirming the important role of the LBP domain in PRRSV replication, which provides a future research direction for the treatment of PRRSV.

## Materials and Methods

### Ethics Statement

All experimental protocols were conducted in accordance with the Guidelines for the Care and Use of Experimental Animals and approved by the Ethical Committee of the Shanghai Veterinary Research Institute, Chinese Academy of Agricultural Sciences (SHVRI-CAAS) (number SHVRI-SZ-20190602-02).

### Cells and Virus

SP2/0 myeloma cells were cultured in Dulbecco's modified Eagle's medium (Gibco) containing 15% fetal bovine serum (FBS). AMs were prepared from the lung lavage fluid of 6-week-old healthy PRRSV-free piglets and cultured in RPMI 1,640 medium (Sigma) containing 10% heat-inactivated FBS at 37°C under 5% CO_2_. Highly pathogenic PRRSV (HP-PRRSV) strain HuN4 labeled with EGFP, HuN4-EGFP, was stored in our laboratory, and the characteristics of HuN4-EGFP were consistent with HuN4 (GenBank accession no. EF635006) (14).

### Expression and Purification of His-Fused CD163 SRCR5-6 Domain

Primers were designed according to the nucleotide sequence of porcine CD163 (GenBank accession no. EU016226). The SRCR5-6 domain of the CD163 was amplified with the primers and inserted into the vector pCold-I, and the positive plasmid was transformed into *Escherichia coli* BL21(DE)3. An aliquot (0.2 mL) of an overnight *E. coli* culture harboring the plasmid was diluted to 200 mL with Luria-Bertani (LB) broth and incubated until the mid-log phase (optical density [OD] ~0.5) at 37°C. Protein expression was then induced with IPTG at a final concentration of 1 mM. After incubation at 16°C for 12 h, the cells were harvested by centrifugation and resuspended in 20 mL of lysis buffer (10 mM imidazole, 20 mM sodium phosphate, 0.5 M NaCl, pH 7.4). The cells were sonicated on ice and centrifuged for 10 min at 10,000 rpm/min. The precipitates and supernatants were separately electrophoresed with 12% SDS-PAGE and visualized with Coomassie Brilliant Blue staining. Then wash off the impure protein of the inclusion bodies with buffer I (2 M urea, 0.1% Triton 100, 50 mM NaCl, 0.2 mM EDTA, PBS, PH 7.4) and buffer II (2 M urea, 0.1% Triton100, PBS) for 10 min at 12,000 rpm/min successively, and dissolved the inclusion bodies with 1 mL Elution Buffer (8 M urea, 0.5 M NaCl, 20 mM NaHPO4, 20 mM NaH2PO4). An anti-His mAb was used to verify antigenic specificity of the purified protein by Western blotting.

### Mouse Immunization Procedure

Five specific-pathogen-free mice were immunized subcutaneously with 100 ng of purified recombinant His-tagged CD163 SRCR5-6 protein emulsified in complete Freund's adjuvant. The mice were then given three further injections of the antigen mixed with incomplete Freund's adjuvant at 14-day intervals.

### mAbs Screening

Splenocytes of an immunized mouse were isolated 2 weeks after the last immunization. The splenocytes were fused with the SP2/0 myeloma cells with polyethylene glycol 1450 (Sigma, USA). The fused cells were cultured in trophoderm-containing hypoxanthine-aminopterin-thymidine (HAT) medium and 15% FBS. The supernatants were collected for an indirect enzyme-linked immunosorbent assay (ELISA) to select positive mAb-producing hybridomas. The positive colonies were then sub-cloned more than three times with a limiting dilution assay. Female BALB/c mice were immunized with the positive clones by intra-peritoneal injection, which pre-injected with pristane to obtain ascites fluid.

### Indirect ELISA

Ninety-six-well microplates were coated with 100 ng/well CD163 SRCR5-6 in carbonate coating buffer (pH 9.6) overnight at 4°C. The plates were then washed three times with PBST (1 × PBS, 0.1% Tween 20; 10 min per wash) and blocked with 5% skimmed milk at for 2 h at 37°C. After the plates were washed three times with PBST, 100 μL of the hybridoma supernatant, positive antiserum, or negative antiserum (blank control mouse serum) was added to each well and incubated for 1 h at 37°C. After the plates were washed with PBST, a 1:10,000 dilution of HRP-conjugated goat anti-mouse IgG antibody in 5% skimmed milk was added to each well and the plates were incubated for 1 h at 37°C. After three washes, 50 μL of 3,3′,5,5′-tetramethylbenzidine (TMB) liquid (Amresco, Solon, Ohio, USA) was added to each well and the plates were incubated for 12 min at room temperature. The reaction was stopped by the addition of 50 μL of 2 M H_2_SO_4_. The OD values were measured immediately at a wavelength of 450 nm (OD_450_).

### Mapping the B-Cell Epitopes With Monoclonal Antibodies

The CD163 SRCR5 domain and SRCR6 domain are located between amino acids 477–482 of CD163. Sequences encoding CD163 amino acids 477–545, 535–614, and 604–682 were cloned into the pCold-TF vector after amplification with the primers shown in Table 1. Then transform these plasmids into BL21 (DE) 3 for expression.

**Table 1:** Primer for plasmids construction.

**Primer**^**a**^	**Sequence** (5^**′**^-3^**′**^)^**b**^
pColdI-477-682aa-F	TCGGTACCCTCGAGGGAATGCCCAGGCTGGTTGGAGGGGAC
pColdI-477-682aa-R	AGCTTGAATTCGGATCTCATGAGCAGATTACAGAGGCCAC
pColdI-51-1116aa-F	CTCGAGGGATCCGAAATGCTGAGGCTAACGGGTGGTG
pColdI-51-1116aa-R	GTCGACAAGCTTGAATTTCATTGTACTTCAGAG
pCold-TF-477-545aa-F	AGGCATATGGAGCTCGGTACCCCCAGGCTGGTTGGAGGG
pCold-TF-477-545aa-R	TTCGGATCCCTCGAGGGTACCTCACTGGAATTCTTCAGCCCAGATC
pCold-TF-535-614aa-F	AGGCATATGGAGCTCGGTACCAGTGGACAGATCTGGGCTGAAG
pCold-TF-535-614aa-R	TTCGGATCCCTCGAGGGTACCTCATTCCATGTCCCAGTGAGAGTTGC
pCold-TF-604-682aa-F	AGGCATATGGAGCTCGGTACCTCCCTCTGCAACTCTCACTGGG
pCold-TF-604-682aa-R	TTCGGATCCCTCGAGGGTACCTCATGAGCAGATTACAGAGGCCACTT
pCold-TF-477-506aa-F	AGGCATATGGAGCTCGGTACCCCCAGGCTGGTTGGAGGG
pCold-TF-477-506aa-R	TTCGGATCCCTCGAGGGTACCTCAGAAGTCAGAATCACAGACGGTGC
pCold-TF-487-516aa-F	AGGCATATGGAGCTCGGTACCTCTGGTCGTGTTGAAGTACAACATG
pCold-TF-487-516aa-R	TTCGGATCCCTCGAGGGTACCTCACCTGCACAGCACGCTGGC
pCold-TF-497-526aa-F	AGGCATATGGAGCTCGGTACCACGTGGGGCACCGTCTGT
pCold-TF-497-526aa-R	TTCGGATCCCTCGAGGGTACCTCAGAGGGAAACCACAGTGCCG
pCold-TF-507-536-aa-F	AGGCATATGGAGCTCGGTACCTCTCTGGAGGCGGCCAGC
pCold-TF-507-536-aa-R	TTCGGATCCCTCGAGGGTACCTCAACTTCCTTCTCCAAAGTGAGCTCC

a *F denotes forward PCR primer; R denotes reverse PCR primer*.

b *Restriction sites, and homologous arm are underlined*.

To further map the epitope based on the first mapping results, a series of plasmids was constructed in pCold-TF with the primers shown in Table 1. *E. coli* BL21(DE3) cells were then transformed with these plasmids and the encoded peptides expressed. All the samples were separated electrophoretically with 12% SDS-PAGE and incubated with an anti-His antibody or the hybridoma supernatant.

To exactly map the epitope, several peptides were synthesized by Shanghai GL Peptide Ltd. ELISA plates were coated with the synthesized peptides (400 ng/well) in carbonate bicarbonate buffer (15 mM Na_2_CO_3_, 35 mM NaHCO_3_, pH 9.6) and incubated overnight at 4°C. The plates were blocked with 5% skim milk in phosphate buffer containing 0.05% Tween 20 for 1 h at 37°C.

After the plates were washed three times, they were incubated at 37°C for 1 h with 100 μL of supernatant from the hybridoma cells or diluted antiserum. The plates were washed three times in PBST and incubated at 37°C for 1 h with HRP-conjugated goat anti-mouse IgG antibody (Proteintech Group, China) diluted 1:10,000 in PBST. The plates were then washed with PBST and incubated with 50 μL/well TMB liquid for 15 min at room temperature in the dark. After the reaction was stopped with 2 M H_2_SO_4_ (50 μL/well), the results were read as OD450 values (15).

### Western Blotting

Cell lysates were prepared with RIPA buffer supplemented with protease inhibitors and phosphatase inhibitors. Equal amounts of cell lysates were fractionated with SDS-PAGE and then blotted onto nitrocellulose membranes. The membranes were blocked for 1 h at room temperature with 5% skim milk and then probed with mAb 1D7 supernatant for 2 h at room temperature. After the membranes were washed with TBST (10 min each), they were incubated with an HRP-conjugated goat anti-mouse antibody (1:6000; Proteintech) for 1 h at room temperature. After the membranes were washed three times, the signals were visualized with an ECL kit (Thermo Fisher) and detected with the Tanon 5200 Chemiluminescent Imaging System.

### Identification of mAb Subtype

The supernatants of hybridoma cells were diluted 1:100, according to the introduction of the mouse monoclonal antibody subtype identification kit (Proteintech). The diluted supernatants of hybridoma cells were added to the plate(50 μL/well) and then mixed with 50 μL/well HRP-conjugated goat anti-mouse IgA+IgM+IgG antibodies. The plate was covered with sealing film and incubated for 1 h at room temperature. TMB (100 μL/well) was added to the plate, which was incubated for 10–20 min at room temperature in the dark. Stop solution (100 μL) was added to each well to stop the reaction. The OD_450_ values were then read and recorded.

### Virus Blocking Test

Porcine AMs were cultured in a 24-well plate. Each group of cells included three repetitions, and the contents of three wells were mixed for RNA extraction. The AMs of different groups were pre-treated with or without different concentrations of 1D7 ascitic fluids for 2 h at 37°C. The cells were then infected with recombinant HP-PRRSV HuN4-EGFP at a multiplicity of infection (MOI) of 0.005, and incubated for 48 h at 37°C. The supernatants were cast off and the cells washed three times. The RNA was extracted from the cells with the Qiagen RNeasy^®^ Mini Kit (Qiagen) and reverse transcribed into cDNA with TaKaRa PrimeScript RT Master Mix. Absolute quantitative real-time PCRs were performed in triplicate to determine the expression of ORF7 gene on transcription level. And TCID50 were also used to analysis the viral infection and replication.

### Statistical Analysis

All results are representative of three independent experiments. Statistical analyses were performed with the GraphPad Prism 5.0 software (GraphPad). Significance was determined with Student's two-tailed *t*-test. Statistical significance: ^*^p < 0.05, ^**^p < 0.01, ^***^p < 0.001.

## Results

### Preparation of Recombinant Proteins

**Figure 1:**
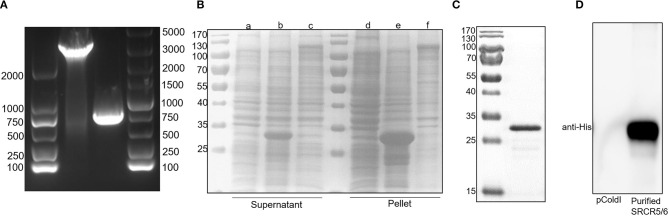
Expression of His-tagged CD163 SRCR5-6 protein and its purification with SDS-PAGE. **(A)** Amplification of CD163 nucleotides 151-3348 and CD163 SRCR5-6. **(B)** Purification of pCold-I-SRCR5-6 with SDS-PAGE. Lanes a-c contain the supernatants of cells expressing pCold-I, pCold-I-SRCR5-6, and pCold-I-CD163, respectively, induced with IPTG, after ultrasonication. Lanes d-f contain the pellets of cells expressing pCold-I, pCold-I-SRCR5-6, and pCold-I-CD163, respectively, induced with IPTG, after ultrasonication. **(C)** Analysis of purified of His-tagged CD163 SRCR5-6 protein with SDS-PAGE. **(D)** Identification of purified His-tagged CD163 SRCR5-6 protein with western blotting.

Full-length CD163 and CD163 SRCR5-6 was amplified from cDNAs of total RNA of porcine AM (Figure 1A). However, the protein expression level of full-length CD163 was lower than that of CD163 SRCR5-6 (Figure 1B), not enough to generate sufficient antigen to induce a robust immune response. Because CD163 SRCR5 and SRCR6 are relatively conserved and are important for PRRSV infection, a recombinant His-fused CD163 SRCR5-6 protein was successfully expressed in *E. coli* BL21(DE3) cells (Figure 1B). The protein was expressed both in the cell supernatant and as inclusion bodies, but the His-fused CD163 SRCR5-6 protein was mainly present as inclusion bodies. The protein was then purified with urea gradient centrifugation. A western blotting analysis showed that the purified His-fused CD163 SRCR5-6 protein was identified by an anti-His mAb (Figures 1C,D).

### Development of mAbs Against CD163 SRCR5-6

**Figure 2:**
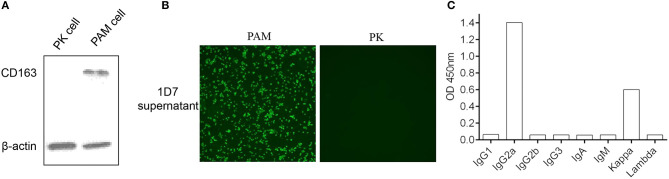
Confirmation of the specificity of mAb 1D7. **(A)**. 1D7 was used for the analysis of endogenous CD163 expression of porcine AMs and PK cells with western blotting. **(B)** Immunofluorescence was used to analysis the specific of 1D7 mAb. **(C)**. Subtype identification of 1D7 with a mouse antibody subtype identification kit.

Five female BALB/c mice were separately immunized with the purified His-fused CD163 SRCR5-6 protein to generate hybridomas producing specific antibodies against CD163 SRCR5-6. A hybridoma clone (1D7) was identified by screening the supernatants with a CD163-SRCR5-6-specific indirect ELISA. CD163 is a molecular marker of macrophage maturation. The expression of CD163 in AMs is relatively high, whereas CD163 is completely absent from PK-15 cells. To verify the specificity of the mAb, the lysis of porcine AMs and PK-15 cells were used in Western Blotting (Figure 2A) and an immunofluorescence assay (Figure 2B). The results showed that 1D7 only reacted with the lysis of porcine AMs and could be used to detect endogenous CD163. The mAb subtype was then identified with a mouse monoclonal antibody subtypes detection kit (Proteintech). The heavy chain of 1D7 was IgG2a, whereas the light chain was a kappa chain (Figure 2C).

### Epitope Identification of mAb 1D7

**Figure 3:**
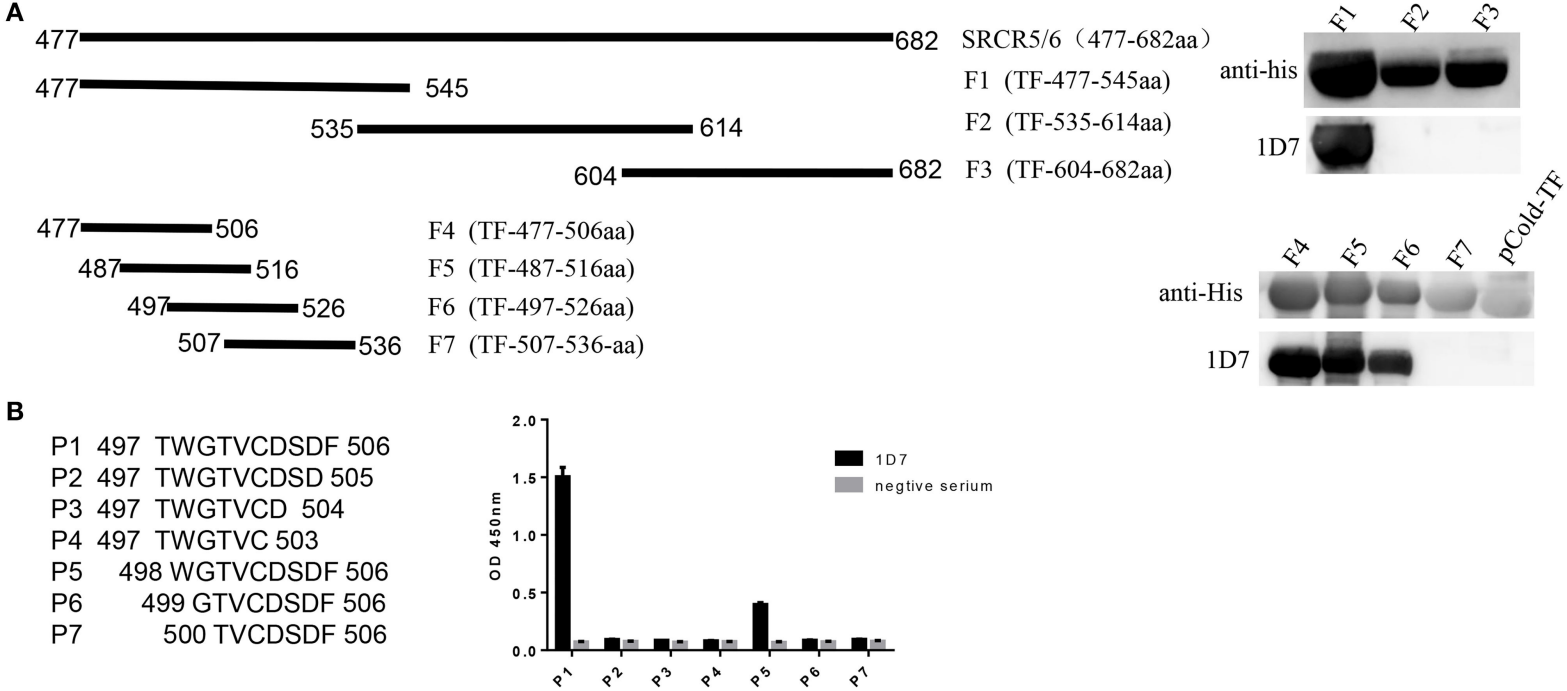
Mapping the epitope of mAb 1D7. **(A)** A series of truncated SRCR5-6 were constructed to pCold-TF and was successfully expressed in *E. coli* BL21(DE3) cells. Protein expression was then induced with IPTG. After incubation at 16°C for 12 h, the cells were harvested for SDS-PAGE. Schematic diagram and western blotting results using 1D7 to probe truncated TF-SRCR5-6. **(B)** A battery of peptides corresponding to amino acids 497–506 were synthesized. Four hundred nano grams peptides were coated to the ELISA plates in order. Indirect ELISA was used to analysis the epitope of 1D7.

To identify the epitope recognized by the mAb, three overlapping fragments (F1, F2, F3) comprising partial length of CD163 SRCR5-6, were cloned into pCold-TF vector and the positive recombinant plasmids were transformed into *E. coli* BL21(DE)3 and induced by IPTG, and analyzed with Western blotting. The results showed that the epitope recognized by 1D7 was located between amino acids 477 and 535 (Figure 3A). Another four overlapping TF-tagged peptides (F4, F5, F6, F7) spanning amino acids 477–535 were then expressed. The epitope was within the region defined by amino acids 497–506 (Figure 3A). To confirm the minimal linear epitope recognized by 1D7, a series of peptides (P1–P7) spanning amino acids 497–506 of CD163 SRCR5-6 was synthesized by Shanghai GL Peptide Ltd. Based on the results of ELISA, we concluded that the minimal linear epitope recognized by 1D7 is ^497^TWGTVCDSDF^506^ (Figure 3B).

### mAb 1D7 Could Partially Block PRRSV Replication

**Figure 4:**
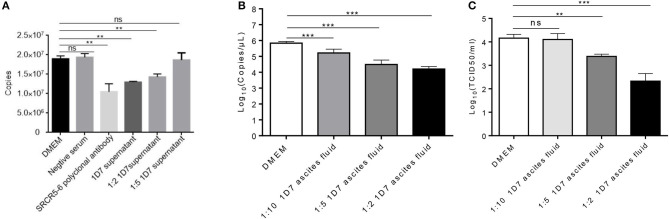
Role of CD163 antibody 1D7 in blocking PRRSV infection. **(A)** Porcine AMs were pre-treated with different doses of 1D7 supernatant before PRRSV infection for 2 h at 37°C. The cells were then infected with HP-PRRSV HuN4, and incubated for 2 h at 37°C. The total RNA of AMs were extracted, and RT-qPCR was used to detect the transcription level of ORF7 mRNA. **(B)** Porcine AMs were pre-treated with different doses of 1D7 ascites fluid for 2 h at 37°C followed by HuN4-EGFP infection at a MOI of 0.005 for 48h at 37°C; the cell supernatants were then collected, and the total RNA of supernatants were extracted according to M&M. RT-qPCR was used to identify viral replication. **(C)** TCID_50_ was also used to identify viral replication. Error bar: mean ± SEM; **p ≤ 0.01; ***p ≤ 0.001.

To verify the neutralization activity of 1D7, the recombinant HuN4-EGFP was used. RT-qPCR, TCID_50_ were used to detect the viral infection and replication. The number of ORF7 copies was lower in the infected cells pre-treated with both 1D7 supernatant and ascites fluid than those not treated with 1D7 (Figures 4A–C). At higher concentrations of 1D7, the expression of the viral replication was further reduced, indicating a dose-dependent relationship. The repeatability of the experiment was good. The mAb exerted some degree of viral blocking, but the effect was not very marked. We infer that the mAb is directed against the linear epitope ^497^TWGTVCDSDF^506^. Its blocking effect on viral infection was limited compared with the effects of polyclonal antibody with conformational epitopes (16).

## Discussion

PRRSV is characterized by high antigenic variability, so there is still no effective prevention or control measures for PRRSV infection. Studies of the PRRSV receptors may offer new avenues of research for its prevention and control. Many studies have confirmed that CD163 is an essential receptor for PRRSV on its host cells, and has been shown to interact with PRRSV proteins GP2a and GP4, facilitating the uncoating and release of the viral genome into the cytoplasm at the low pH within the early endosome (7). CD163-knockout pigs were fully resistant to HP-PRRSV (17), and pigs in which the SRCR5 domain of CD163 was deleted fully resisted PRRSV infection, further studies showed that pigs in which a 41-amino-acid fragment (amino acids 481–521) containing the LBP motif (amino acids 487–495) in the SRCR5 domain of CD163 deleted were fully resistant to PRRSV *in vivo* (18). Many researchers have focused on the CD163, indicating that SRCR5 deletion affects the uncoating of PRRSV rather than its entry, and overexpression of ADAM17 can downregulate the expression of CD163 on cell membranes following a reduction in PRRSV infection, while the inhibition of ADAM17 upregulates the membrane CD163 following a promotion in PRRSV infection, and TREM2 can facilitate PRRSV infection via increasing the expression of CD163 and decreases the cleavage of membrane CD163 mediated by ADAM17 (19, 20). However, many aspects of CD163 still require clarification before the role of CD163 in PRRSV replication can be determined.

At present, the mAb most frequently used in molecular research into pig CD163 is anti-CD163 mAb 2A10/11 which targets the SRCR1–3 (21), produced by Bio-Rad Laboratories. Because 2A10/11 can be used for flow cytometry, immunofluorescence, and immunoprecipitation, it has facilitated considerable research. However, clone 2A10/11 only recognizes porcine CD163 under non-reducing conditions when used for western blotting. This may be inconvenient for samples analyzed with western blotting, especially because such samples are so valuable.

In this study, we generated a mAb 1D7 against CD163 that recognizes porcine CD163 under reducing conditions for usage of western blotting. We also identified a new epitope in the CD163 SRCR5 domain (^497^TWGTVCDSDF^506^). Because the epitope is close to the LBP domain of CD163, there were fewer viral copies in AMs treated with 1D7 than in AMs treated with SP20 supernatant. Although the blocking effect of ascites fluid was better than 1D7 supernatant, it was still limited. In a word. 1D7, the anti-CD163 monoclonal antibody provides a foundation for the analysis of other biological functions of CD163, and the blocking effect of the mAb on PRRSV can provide a research idea and future research direction for the treatment of PRRSV. Therefore, the specific antibody directed against the CD163 SRCR5 domain generated in this study provides a tool for functional studies of CD163 and further investigation into the link between CD163 and viral infection.

## Data Availability Statement

The raw data supporting the conclusions of this article will be made available by the authors, without undue reservation.

## Ethics Statement

The animal study was reviewed and approved by Laboratory Animal Welfare and Ethics Committee of the Shanghai Veterinary Research Institute-SHVRI-SZ-20190602-02.

## Author Contributions

FG and YZha: conceptualization, visualization and, wrote–original draft. YZha and KZhan: data curation and formal analysis. FG and GT: funding acquisition, project administration, resources, and supervision. YZha, KZhan, and CL: investigation. YZha, KZhan, HZ, ND, YZho, LY, GL, and WT: methodology. YZha, LL, and YJ: software. YZha, KZhan, WT, and KZhao: validation. YZha, FG, HZ, and KZhan: wrote–review and editing. All authors: have read and agreed to the published version of the manuscript.

## Conflict of Interest

The authors declare that the research was conducted in the absence of any commercial or financial relationships that could be construed as a potential conflict of interest.
